# Integrative transcriptomic and machine learning analysis identifies PYCARD and IFI30 as immune-lysosomal biomarkers of ANCA-associated glomerulonephritis

**DOI:** 10.1080/0886022X.2026.2624286

**Published:** 2026-02-10

**Authors:** Liyuan Bei, Jing Liao, Zhenhua Yang, Shihua Li, Mu Huang, Ling Lei

**Affiliations:** ^a^ Nephrology Department, The First Affiliated Hospital of Guangxi Medical University, Nanning, Guangxi, China; ^b^ Ruikang Hospital, Affiliated to Guangxi University of Chinese Medicine, Intensive Care Unit, Nanning, Guangxi, China; ^c^ Department of Rheumatology and Immunology, The First Affiliated Hospital of Guangxi Medical University, Nanning, Guangxi, China

**Keywords:** ANCA-associated vasculitis, glomerulonephritis, PYCARD, IFI30, mitophagy, inflammasome

## Abstract

**Objectives:**

ANCA-associated glomerulonephritis (ANCA-GN) is an immune-mediated kidney disease leading to acute or chronic renal failure. This study investigates the role of mitophagy-related genes in ANCA-GN, as mitochondrial dysfunction is closely linked to the pathogenesis of various kidney diseases.

**Methods:**

This study analyzed transcriptomic data from GEO datasets (GSE104948 and GSE108109) to investigate mitophagy-related mechanisms in ANCA-GN. Methods included batch correction, consensus clustering (identifying two subtypes), weighted gene co-expression network analysis (WGCNA), differential expression screening, and machine learning (LASSO, random forest, SVM-RFE). A diagnostic nomogram was constructed and validated, and immune cell infiltration was profiled.

**Results:**

Analyses revealed distinct activation of immune pathways, including complement and phagosome signaling, alongside abnormal infiltration of CD8+ T cells in ANCA-GN. Subtype-specific analysis identified 131 differentially expressed genes (DEGs), while 143 DEGs distinguished ANCA-GN from controls.Intersection analysis and machine learning prioritized two hub genes, *PYCARD* and *IFI30*, which exhibited strong diagnostic accuracy (AUC >0.9) and correlated with CD8+ T-cell infiltration. A nomogram model validated their clinical utility (AUC >0.9). Functional enrichment highlighted phagocytosis and immune signaling pathways. Immune profiling revealed significant upregulation of 20 immune cell types in ANCA-GN.

**Conclusions:**

These findings suggest that mitophagy-immune crosstalk drives ANCA-GN progression, with PYCARD and IFI30 as potential diagnostic biomarkers. This study provides mechanistic insights into ANCA-GN pathogenesis and proposes novel targets for clinical intervention.

## Introduction

ANCA-associated glomerulonephritis (ANCA-GN) is a severe autoimmune renal disorder characterized by necrotizing and crescentic glomerular inflammation, which may progress rapidly to acute or chronic renal failure[1]. According to the *KDIGO 2021 Clinical Practice Guideline for the Management of Glomerular Diseases* (*Kidney Int.* 2021;100(4S):S1–S276), ANCA-GN represents the renal manifestation of ANCA-associated vasculitis and is defined by pauci-immune glomerulonephritis with circulating anti-neutrophil cytoplasmic antibodies. The disease has an annual incidence of approximately 1–20 cases per million population, predominantly affecting middle-aged and elderly individuals (peak age 50–70 years), with an emerging trend toward younger onset and an approximately equal sex distribution. The disease significantly impacts patients’ quality of life and presents a considerable economic burden on healthcare systems due to the high costs associated with renal replacement therapies [2]. Current treatment strategies primarily involve immunosuppressive agents and biological therapies; however, their efficacy could vary widely among patients due to individual differences in disease etiology and progression [3]. Moreover, a notable subset of patients demonstrates inadequate responses to existing treatments, underscoring the urgent need for novel therapeutic strategies and biomarkers to enhance disease management and patient outcomes.

Mitophagy is the selective degradation of damaged or superfluous mitochondria *via* the autophagic–lysosomal pathway, thereby preserving mitochondrial quality and cellular homeostasis [4]. Recent studies have underscored the critical involvement of mitochondrial dysfunction in the pathogenesis of various renal disorders [5]. Although mitochondrial bioenergetics are predominantly essential for the energy-demanding proximal tubular cells, where most evidence has been obtained [6], accumulating data suggest that mitochondrial integrity is also important within the glomerulus [7]. In the renal diseases such as lupus nephritis and diabetic kidney disease, both intrinsic glomerular cells (e.g., podocytes and mesangial cells) and infiltrating immune cells (e.g., macrophages) rely on mitochondrial homeostasis for cytoskeletal stability, redox balance, and immune signalling [8–11]. These findings imply that mitochondrial impairment may also contribute to glomerular injury in ANCA-GN, although its precise role remains poorly defined.

PYCARD (also known as ASC) serves as a central adaptor protein of the NLRP3 inflammasome, bridging NLRP3 and caspase-1 through its pyrin (PYD) and caspase recruitment (CARD) domains to promote inflammasome assembly and inflammatory activation [12]. Previous studies have demonstrated that activation of the NLRP3 inflammasome contributes to the pathogenesis of ANCA-associated vasculitis, metabolic kidney diseases, and systemic vasculitis by activating macrophages, inducing endothelial injury, and amplifying renal inflammation [13–16]. Importantly, PYCARD facilitates the formation of neutrophil extracellular traps (NETs) through activation of the NLRP3 inflammasome [17,18]. The activation of the NLRP3 inflammasome is now recognized as a pivotal event in the development of ANCA-associated vasculitis, suggesting that PYCARD, as the core adaptor of NLRP3, may play an essential regulatory role in renal inflammation and immune responses [14,19,20].

IFI30 (γ-interferon–inducible lysosomal thiol reductase, GILT) functions within lysosomes to promote the reduction and processing of antigens, thereby facilitating MHC class II–dependent presentation of self-antigens such as myeloperoxidase (MPO) and proteinase 3 (PR3) [21]. Upregulation of IFI30 has been observed in systemic autoimmune diseases and renal tissues, where its dysregulated activity may enhance antigen presentation, promote autoantibody production (including ANCA), and exacerbate immune-mediated tissue injury [22–24].

Collectively, these findings suggest that PYCARD and IFI30 may play critical roles in the pathogenesis of ANCA-GN, with PYCARD contributing through mechanisms such as promoting NETosis and activating macrophages [25,26], while IFI30 enhances lysosomal antigen presentation. Their involvement in inflammasome activation and immune–lysosomal processing may intersect with mitochondrial quality control pathways, providing a molecular framework for exploring their potential as diagnostic biomarkers and therapeutic targets in ANCA-GN.

## Materials and methods

This study, which leverages machine learning and genomic data, followed the TRIPOD-AI and STREGA reporting guidelines to ensure transparent and complete reporting [27,28]. The R scripts, processed expression matrices, and primary plotting code generated in this study are available on Zenodo at DOI:10.5281/zenodo.17519865.

### Data sources and download

All data used in this study were publicly available and retrieved from the Gene Expression Omnibus (GEO) database, the sample IDs, phenotype metadata, and batch labels used in the analyses are provided in Supplementary Table 1. Genome-wide expression profiles were obtained using the R package ‘GEOquery’. The combined dataset included 37 ANCA-GN and 27 control samples derived exclusively from glomerular biopsies *via* laser capture microdissection, thereby restricting our analysis to the glomerular compartment and excluding tubular or whole-kidney components. For external validation, dataset GSE108112 (15 ANCA-GN and 6 control samples) was used. Batch effects were corrected using the ‘sva’ package [29], and principal component analysis was applied for validation. Mitophagy-related genes were obtained from Onishi et al. [30] (Supplementary Table 2). All analyses were confined to comparisons between ANCA-GN and healthy controls; no cross-comparison with other forms of glomerulonephritis was performed.

### Consensus clustering

Based on the expression levels of mitochondrial autophagy-related genes, consensus clustering was performed on the ANCA-GN patient samples in the dataset using the R package ‘ConsensusClusterPlus’ [31]. The application of machine learning–based consensus clustering in nephrology has been increasingly validated by a growing body of literature [32–35]. Multiple studies have demonstrated that consensus clustering provides a robust and reproducible framework for uncovering biologically and clinically meaningful patient subgroups across various kidney diseases [36–39].

### Inter-subtype differential analysis

Differentially expressed genes (DEGs) between cluster_1 (*n* = 19) and cluster_2 (*n* = 18) were identified using the R package ‘limma’ (v3.50.0) [40], with thresholds of |log2Fold Change| >1 and adjusted *p*-value < 0.05. Heatmaps were generated using the R package ‘pheatmap’ with Euclidean distance and hierarchical clustering.

### Weighted gene co-expression network analysis (WGCNA)

Co-expression networks were constructed using the R package ‘WGCNA’ [41]. Genes were clustered into modules, and module eigengenes were correlated with mitophagy activity. The turquoise module, showing the strongest association with mitophagy, was selected for further analysis (see Supplementary Materials for network construction details).

### ANCA-GN-specific differential analysis

DEGs between the control group (*n* = 27) and ANCA-GN group (*n* = 37) were identified using the R package ‘limma’ (version 3.50.0) [40], with thresholds of |log2Fold Change| >1.5 and adjusted *p*-value <0.05. Heatmaps were generated using ‘pheatmap’ with Euclidean distance-based hierarchical clustering.

### Gene set enrichment analysis (GSEA)

GSEA is a computational method used to determine whether predefined gene sets show significant and consistent differences between biological states [42]. The GSEA was performed using the R package ‘clusterProfiler (version 4.2.2)’ to rank all genes by their log2Fold Change values, followed by 1,000 gene set permutations. The Molecular Signatures Database (MSigDB) collection, specifically the c2.cp.kegg.v7.5.1.symbols set, was used as the reference gene set [42,43]. Gene sets with a *p*-value less than 0.05 were considered significantly enriched.

### *Gene ontology (GO) and Kyoto encyclopedia of genes and genomes (KEGG) pathway* enrichment

The R package ‘clusterProfiler (version 4.2.2)’ was applied for GO annotation analysis and KEGG pathway enrichment analysis (*p*-value < 0.05) of Mitophagy-related differentially expressed genes [44].

### Receiver operating characteristic (ROC) curve

The R package ‘pROC’ was used to create ROC curves, determine the AUC, and evaluate the diagnostic value of feature genes [45]. An AUC value between 0.5 and 1 indicates the diagnostic performance, with a higher AUC indicating better diagnostic ability.

### Diagnostic nomogram construction and validation

A nomogram for ANCA-GN diagnosis was constructed using the R package ‘rms’. Genes were categorized into high and low expression groups based on their median expression levels. Risk scores were calculated as the sum of individual gene risk scores. The nomogram’s diagnostic value was evaluated using calibration curves and ROC curves. The model was further validated in the GSE108112 dataset by stratifying gene expression into high and low groups and assessing diagnostic performance *via* ROC analysis.

### Immune infiltration analysis

The relative infiltration levels of immune cells in each sample were assessed using single-sample gene set enrichment analysis (ssGSEA). The analysis utilized gene sets representing 28 immune cell types obtained from the Tumor and Immune System Interaction Database (TISIDB) (Supplementary Table 3). ssGSEA Enrichment scores for each immune cell type were calculated based on the gene expression profiles of individual samples. Differences in immune cell infiltration levels between the ANCA-GN and control groups were visualized using the R package ‘ggplot2’ [46].

### Statistical analysis

All statistical analyses were performed using R software (version 4.1.2). Spearman’s rank correlation test was applied to assess associations between two continuous variables. Differences between two groups were evaluated using the Wilcoxon rank-sum test, while comparisons among three or more groups were conducted using the Kruskal-Wallis test. A two-tailed *p*-value <0.05 was considered statistically significant.

## Results

### The workflow of this project is illustrated in the following figure

#### ANCA-GN subtyping and differential analysis

**Figure 1.**
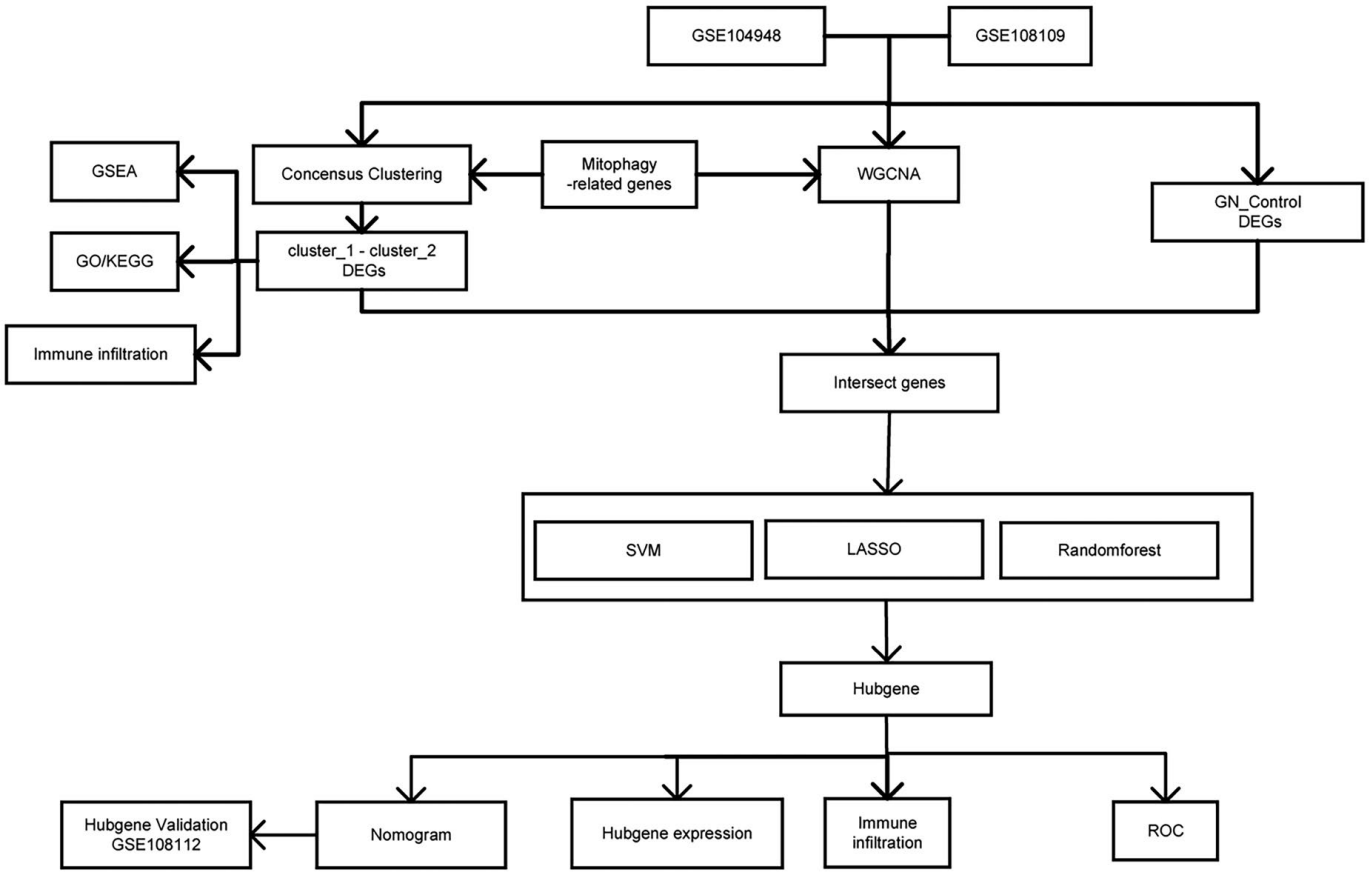
Workflow of the study. GSE: Gene Expression Omnibus Series; GSEA: Gene Set Enrichment Analysis; WGCNA: weighted gene co-expression network analysis; GN: glomerulonephritis; DEGs: differentially expressed genes; GO: Gene Ontology; KEGG: Kyoto Encyclopedia of Genes and Genomes; SVM: Support Vector Machine; LASSO: Least Absolute Shrinkage and Selection Operator; ROC: Receiver Operating Characteristic.

**Figure 2.**
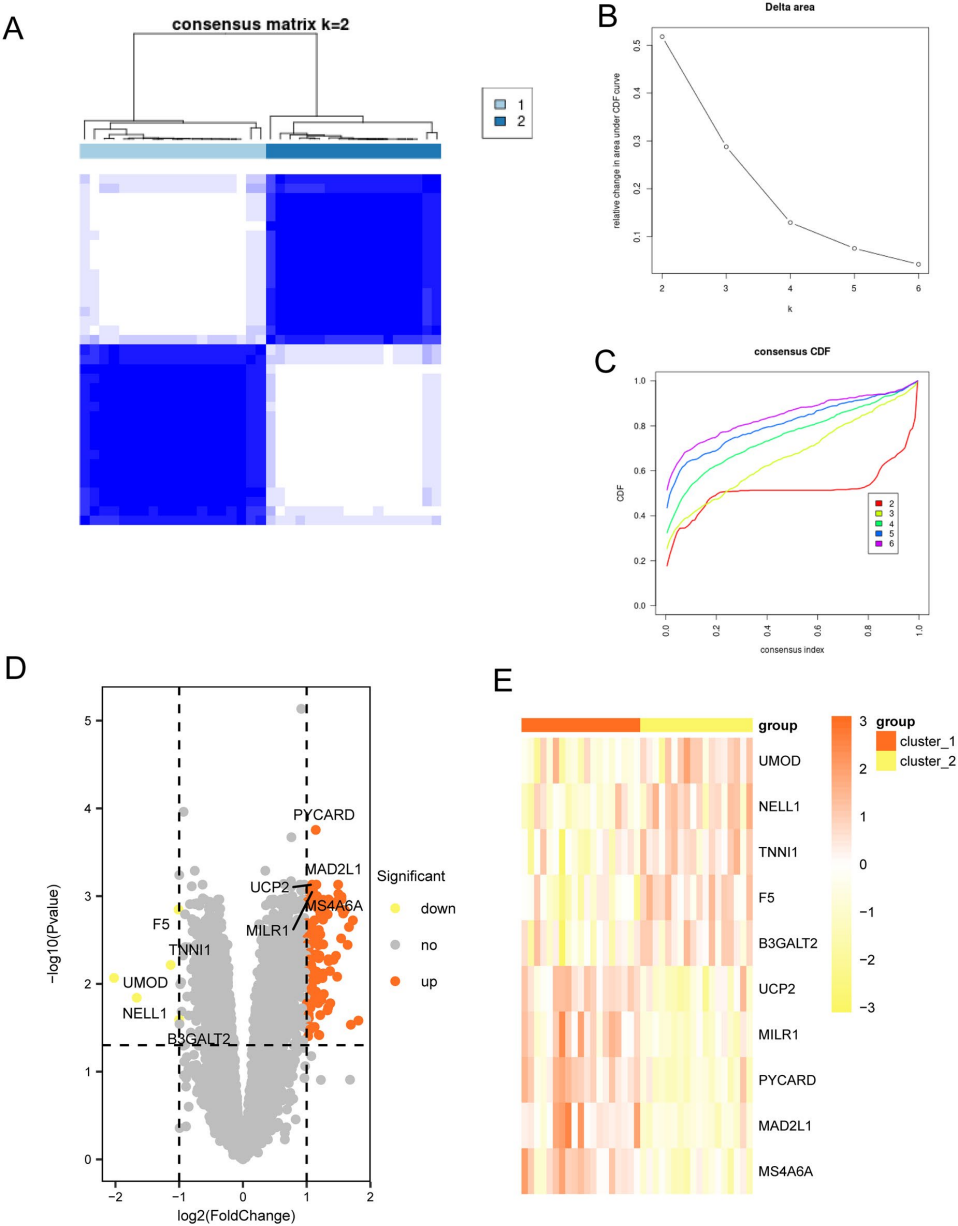
Subtyping and differential analysis based on mitophagy-related genes in ANCA-GN samples. (A) Consensus clustering heatmap for k = 2. The values of the consensus matrix are represented from 0 (unlikely to cluster together) to 1 (always clustering together) with a color gradient from white to dark blue. (B) CDF plot for k values ranging from 2 to 6. The x-axis represents the values of the consensus matrix, and the y-axis represents the cumulative density function. (C) Delta Area plot for k values ranging from 2 to 6. The x-axis represents the number of clusters, and the y-axis represents the relative change in the area under the CDF curve. (D) Volcano plot showing the distribution of DEGs between the cluster_1 and cluster_2 groups. (E) Heatmap depicting the top-ranked DEGs. CDF: Cumulative distribution function; DEGs: differentially expressed genes.

Subtyping of ANCA-GN was performed based on mitophagy-related genes (Figure 1). The optimal number of clusters was determined to be 2, as supported by the consensus clustering heatmap (Figure 2A), CDF curve (Figure 2B), and delta area curve (Figure 2C). Consequently, the ANCA-GN samples were divided into two subtypes: cluster_1 and cluster_2.

A total of 131 DEGs were identified between cluster_1 and cluster_2, with statistical significance (adjusted *p*-value <0.05 and |log2 fold change| >1). Compared to cluster_2,126 genes were upregulated and 5 genes were downregulated in cluster_1 (Supplementary Table 4). The DEGs were visualized using a volcano plot (Figure 2D). Additionally, a heatmap was generated to display the expression patterns of the top-ranked genes *(UCP2, MILR1, PYCARD, MAD2L1, MS4A6A, UMOD, NELL1, TNNI1, F5,* and *B3GALT2)* across the samples (Figure 2E).

### Gene ontology (GO) and Kyoto encyclopedia of genes and genomes (KEGG) pathway enrichment analysis

**Figure 3.**
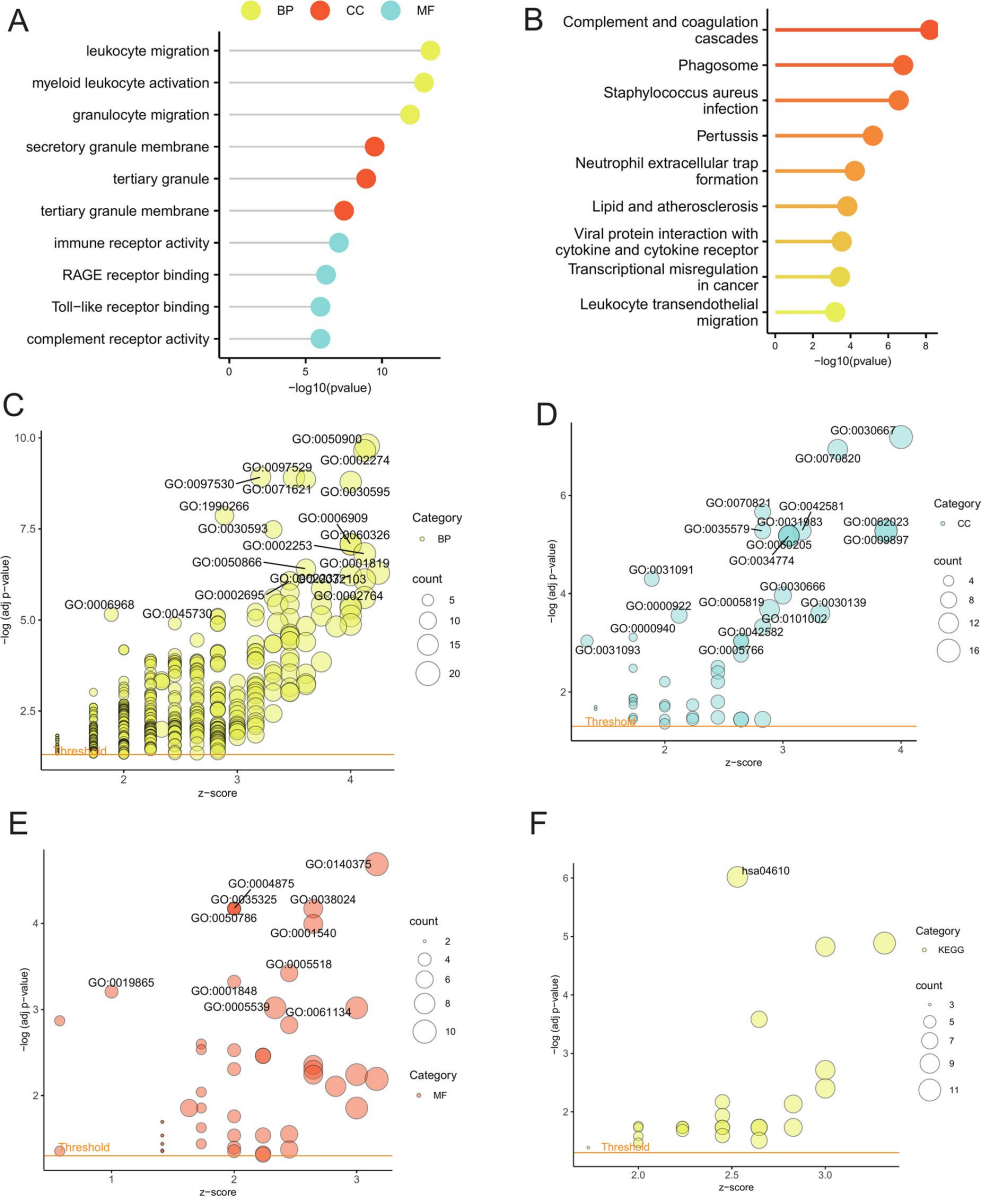
Enrichment analysis based on differentially expressed genes between subtypes. (A) Lollipop plot of GO enrichment terms. (B) Lollipop plot of KEGG enrichment pathways. (C) Bubble plot of BP enrichment terms. (D) Bubble plot of CC enrichment terms. (E) Bubble plot of MF enrichment terms. (F) Bubble plot of KEGG enrichment pathways. GO: Gene Ontology; KEGG: Kyoto Encyclopedia of Genes and Genomes; BP: biological processes; CC: cellular components; MF: molecular functions.

To investigate the biological functions of differentially expressed genes between subtypes, we performed GO term (Supplementary Table 5) and KEGG pathway (Supplementary Table 6) enrichment analyses. The GO results revealed that these genes were enriched in the following biological processes (BP): leukocyte migration (GO:0050900), myeloid leukocyte activation (GO:0002274), granulocyte migration (GO:0097530); cellular components (CC): secretory granule membrane (GO:0030667), tertiary granule (GO:0070820), tertiary granule membrane (GO:0070821); and molecular functions (MF): immune receptor activity (GO:0140375), RAGE receptor binding (GO:0050786), complement receptor activity (GO:0004875), Toll-like receptor binding (GO:0035325) (Figure 3A,C–E). Enriched KEGG pathways included complement and coagulation cascades (hsa04610), phagosome (hsa04145), and Staphylococcus aureus infection (hsa05150) (Figure 3B,F).

### Gene set enrichment analysis

**Figure 4.**
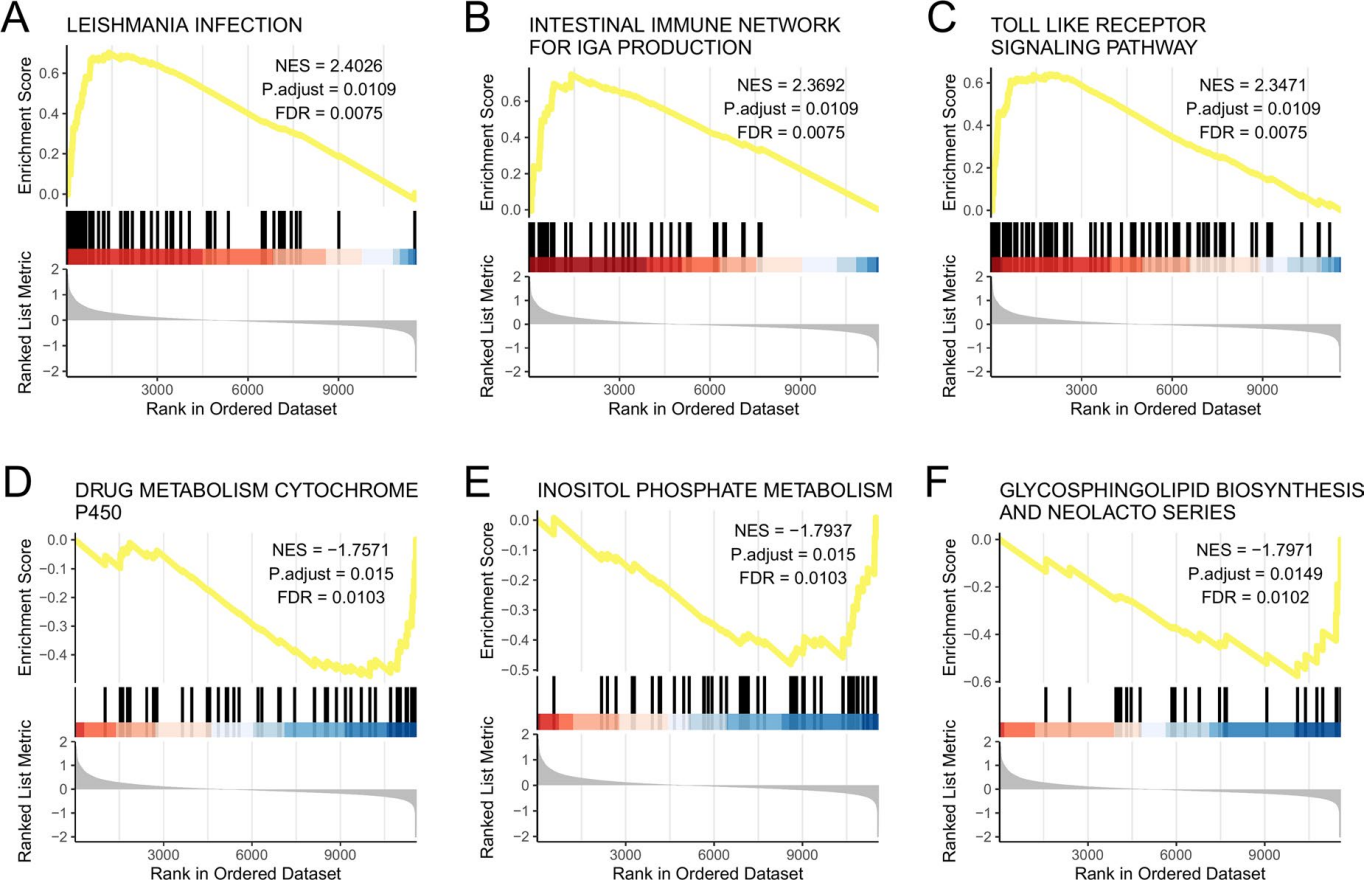
GSEA enriched pathways display. (A) Leishmania Infection. (B) Intestinal Immune Network For IgA Production. (C) Toll-like receptor signaling pathway. (D) Drug Metabolism Cytochrome P450. (E) Inositol Phosphate Metabolism. (F) Glycosphingolipid Biosynthesis Lacto And Neolacto Series.

To further explore the potential mechanisms of differentially expressed genes, we conducted Gene Set Enrichment Analysis (GSEA). Using the MSigDB dataset, we selected the most significantly enriched signaling pathways based on their normalized enrichment score (NES) (Supplementary Table 7). The GSEA revealed significant enrichment in 6 pathways: Leishmania Infection (NES = 2.403, adjusted *p* = 0.011,FDR = 0.008), Intestinal Immune Network For IgA Production(NES = 2.369, adjusted *p* = 0.011, FDR = 0.008), Toll-like receptor signaling pathway (NES = 2.347, adjusted *p* = 0.011,FDR =0.008), Drug Metabolism Cytochrome P450 (NES = −1.757, adjusted *p* = 0.015, FDR = 0.01), Inositol Phosphate Metabolism (NES = −1.794, adjusted *p* = 0.015, FDR = 0.01), Glycosphingolipid Biosynthesis Lacto And Neolacto Series (NES = −1.797, adjusted *p* = 0.015,FDR = 0.01) (Figure 4A–F).

### Construction of weighted gene co-expression network and module identification

**Figure 5.**
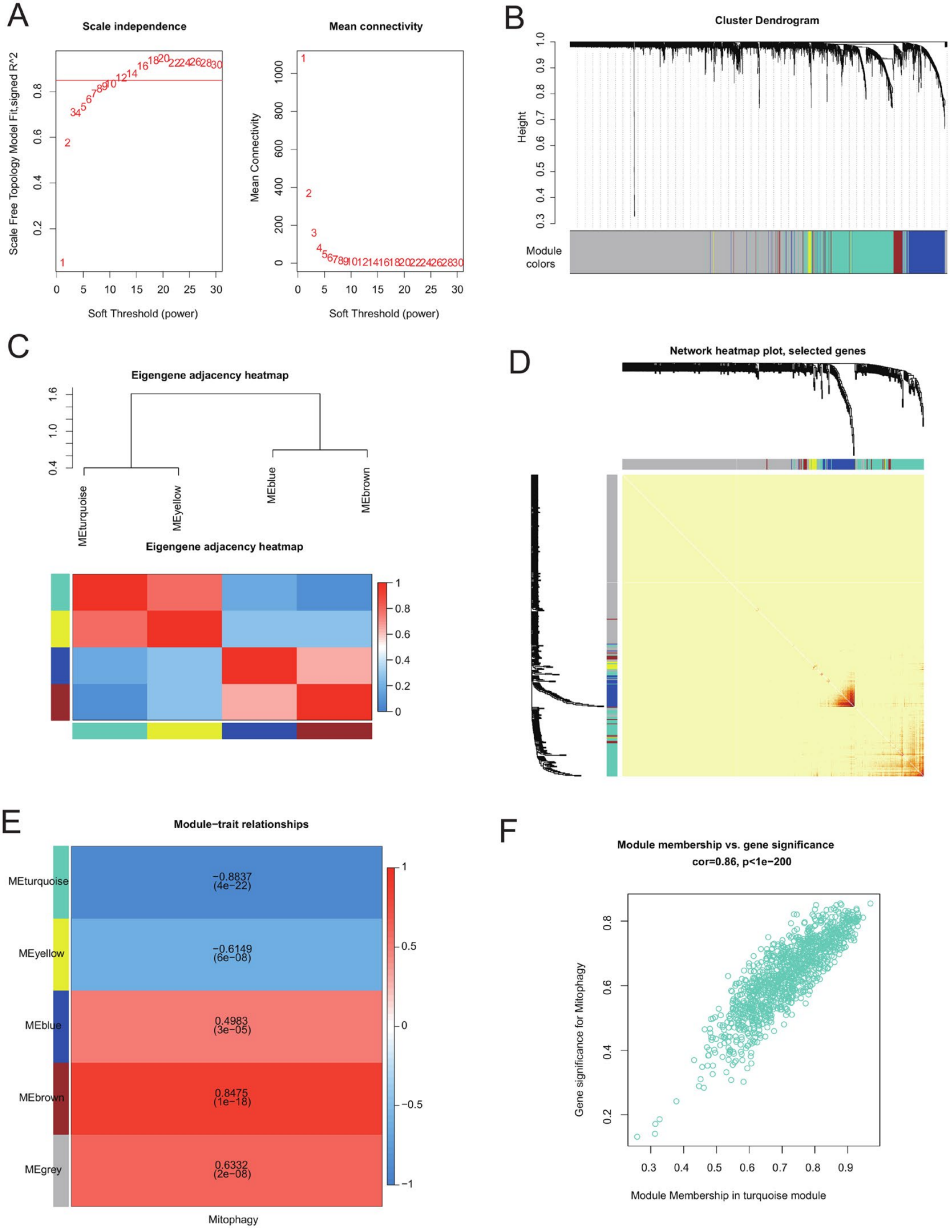
Construction of weighted gene co-expression network analysis co-expression network. (A) Soft threshold β = 12 and the scale-free topology fitting index (R^2^). (B) Co-expression network analysis of genes in ANCA-GN identified different modules of co-expression. (C) Relationship between modules: Correlation heatmap of feature gene networks. Each row and column in the heatmap corresponds to a feature gene of a module (marked by color). Red indicates high adjacency, and blue indicates low adjacency. The red squares along the diagonal represent the meta-modules. (D) Heatmap of topological overlap in the gene network. Each row and column corresponds to a gene, with lighter colors representing low topological overlap and darker red colors indicating high topological overlap. Darker squares along the diagonal correspond to modules. The gene dendrogram and module assignments are displayed on the left and top, respectively. (E) Relationship between consistent module feature genes and mitophagy. Each row in the table corresponds to a consistent module, and each column corresponds to a feature. The numbers in the table represent the correlation between the module feature genes and traits, with P-values shown in parentheses below the correlation values. The correlations are color-coded according to the color legend. (F) Correlation between module membership (MM) and gene significance (GS) for mitophagy in the turquoise module. ‘Cor’ represents the absolute correlation coefficient between GS and MM.ANCA-GN: ANCA-associated glomerulonephritis; MM: module membership; GS: gene significance.

WGCNA was applied to explore gene sets related to mitophagy. Analysis of scale independence and average connectivity revealed that a soft threshold (β) of 12 (Figure 5A) resulted in an average connectivity close to 0 and scale independence >0.85. Four co-expression modules were identified, with uncorrelated genes assigned to the gray module (Figure 5B). We correlated the module eigengenes (MEs) to examine inter-module relationships and used a heatmap to visualize the feature gene network (Figure 5C) and topological overlap (Figure 5D). The four MEs were correlated with mitophagy to identify significant associations. Genes in the turquoise module (*n* = 1133, Supplementary Table 8) showed the strongest negative correlation with mitophagy (*r* = −0.8837, *p* < 0.05) (Figure 5E). Therefore, we focused on the turquoise module, which might best reflect mitophagy. The scatter plot in Figure 5(F) shows a significant positive correlation (cor = 0.86, *p* < 0.05) between gene significance (GS) for mitophagy and module membership (MM), indicating that central genes in the turquoise module are strongly associated with mitophagy.

### Differentially expressed genes associated with ANCA-GN

**Figure 6.**
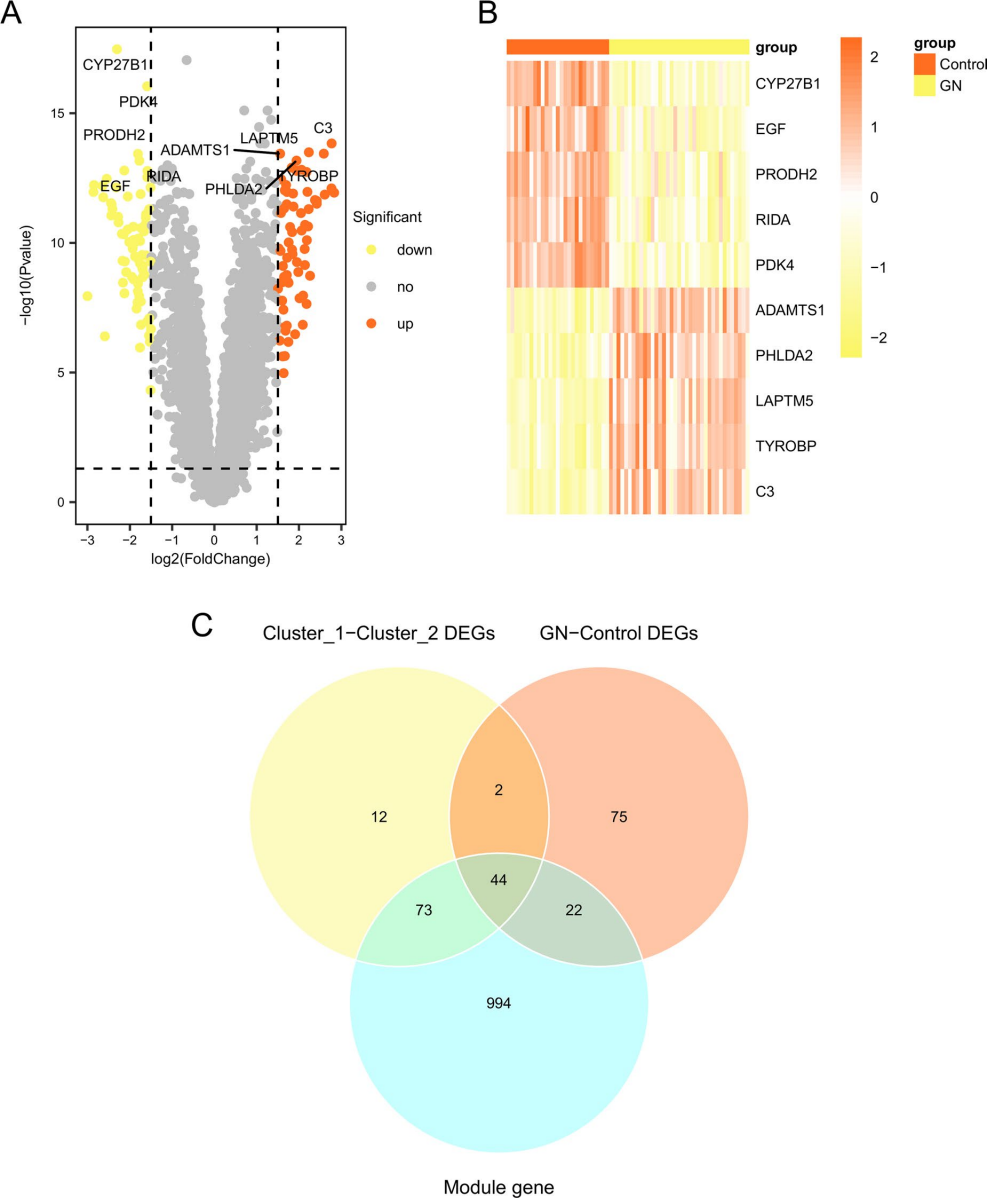
Differential gene analysis related to ANCA-GN. (A) The volcano plot illustrates the distribution of differentially expressed genes (DEGs) between ANCA-GN and control group samples. (B) The heatmap depicts the top-ranked DEGs. (C) The Venn diagram shows the intersection of differentially expressed genes between the subtypes and genes from the most correlated modules in WGCNA with ANCA-GN-related DEGs. ANCA-GN: ANCA-associated glomerulonephritis GN: glomerulonephritis DEGs: differentially expressed genes.

A total of 143 DEGs were identified through comparison between ANCA-GN samples and the control group. These genes showed statistically significant differences between the two groups (adjusted *p*-value < 0.05, |Log2 fold change| > 1.5). Compared to the control group, 67 genes were upregulated and 76 genes were downregulated in ANCA-GN samples (Supplementary Table 9). All DEGs were visualized using a volcano plot (Figure 6A). Additionally, a heatmap displaying the expression levels of the top-ranked genes (*ADAMTS1, PHLDA2, LAPTM5, TYROBP, C3CYP27B1, EGF, PRODH2, RIDA, and PDK4*) in the samples is shown in Figure 6(B). The intersection of differentially expressed genes between subtypes and the most correlated WGCNA module genes with ANCA-GN-related DEGs revealed 44 overlapping genes (Supplementary Table 10).

### Gene ontology (GO) and Kyoto encyclopedia of genes and genomes (KEGG) pathway enrichment analysis

**Figure 7.**
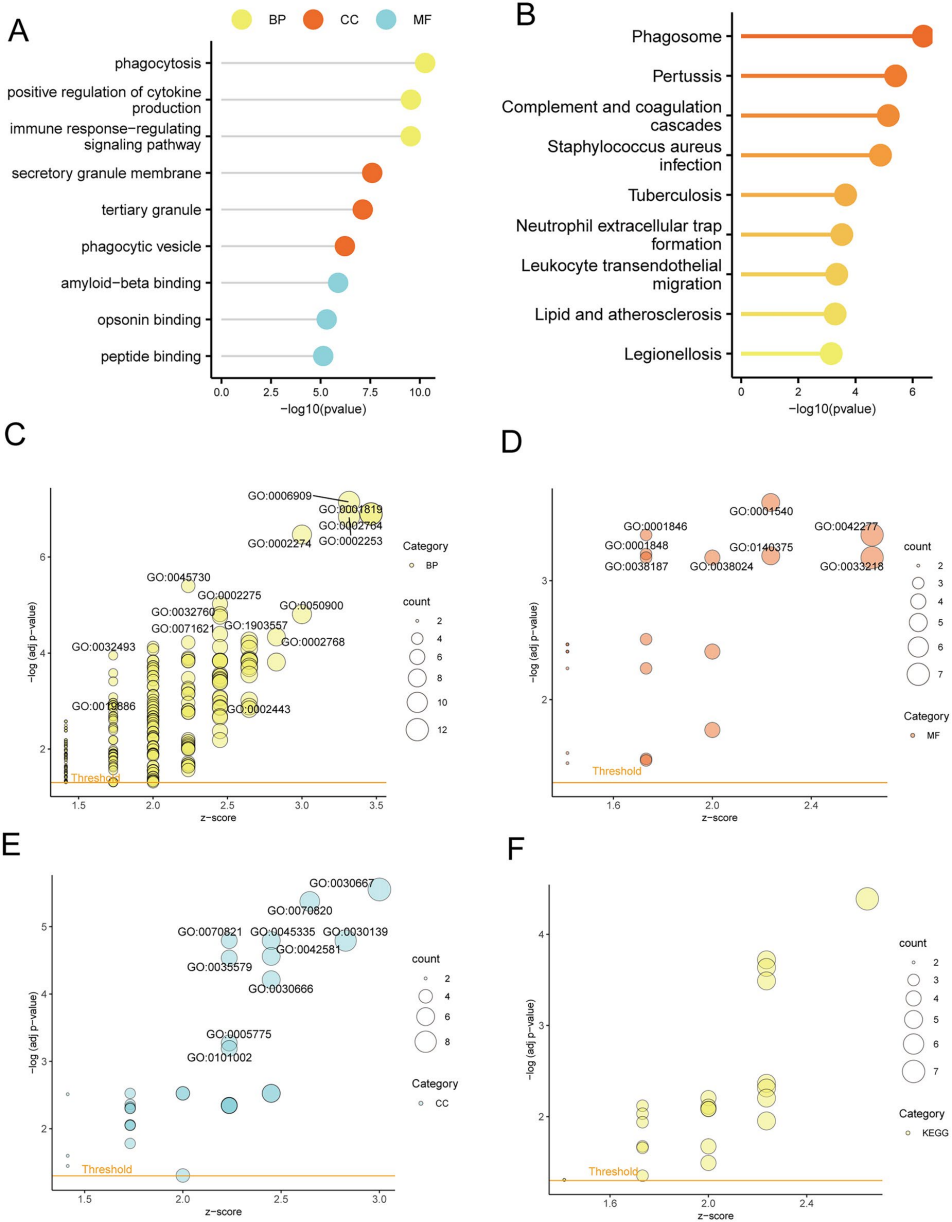
Enrichment analysis based on intersecting genes. (A) The lollipop plot displays the GO enrichment terms. (B) The lollipop plot shows the KEGG enrichment pathways. (C) The bubble plot illustrates the enrichment terms for BP. (D) The bubble plot shows the enrichment terms for MF. (E) The bubble plot presents the enrichment terms for CC. (F) The bubble plot displays the KEGG enrichment pathways. BP: Biological Processes; MF: Molecular Functions; CC: Cellular Components; GO: Gene Ontology; KEGG: Kyoto Encyclopedia of Genes and Genomes.

To investigate the biological functions of the intersecting genes, we performed GO term (Supplementary Table 11) and KEGG pathway (Supplementary Table 12) enrichment analyses. The GO results revealed that these genes were enriched in the following biological processes (BP): phagocytosis (GO:0006909), positive regulation of cytokine production (GO:0001819), immune response-regulating signaling pathway (GO:0002764); cellular components (CC): secretory granule membrane (GO:0030667), tertiary granule (GO:0070820), phagocytic vesicle (GO:0045335); and molecular functions (MF): amyloid-beta binding (GO:0001540), opsonin binding (GO:0001846), peptide binding (GO:0042277) (Figure 7). Enriched KEGG pathways include phagosome (hsa04145), pertussis (hsa05133), and complement and coagulation cascades (hsa04610) (Figure 7).

### Identification of key genes using machine learning

**Figure 8.**
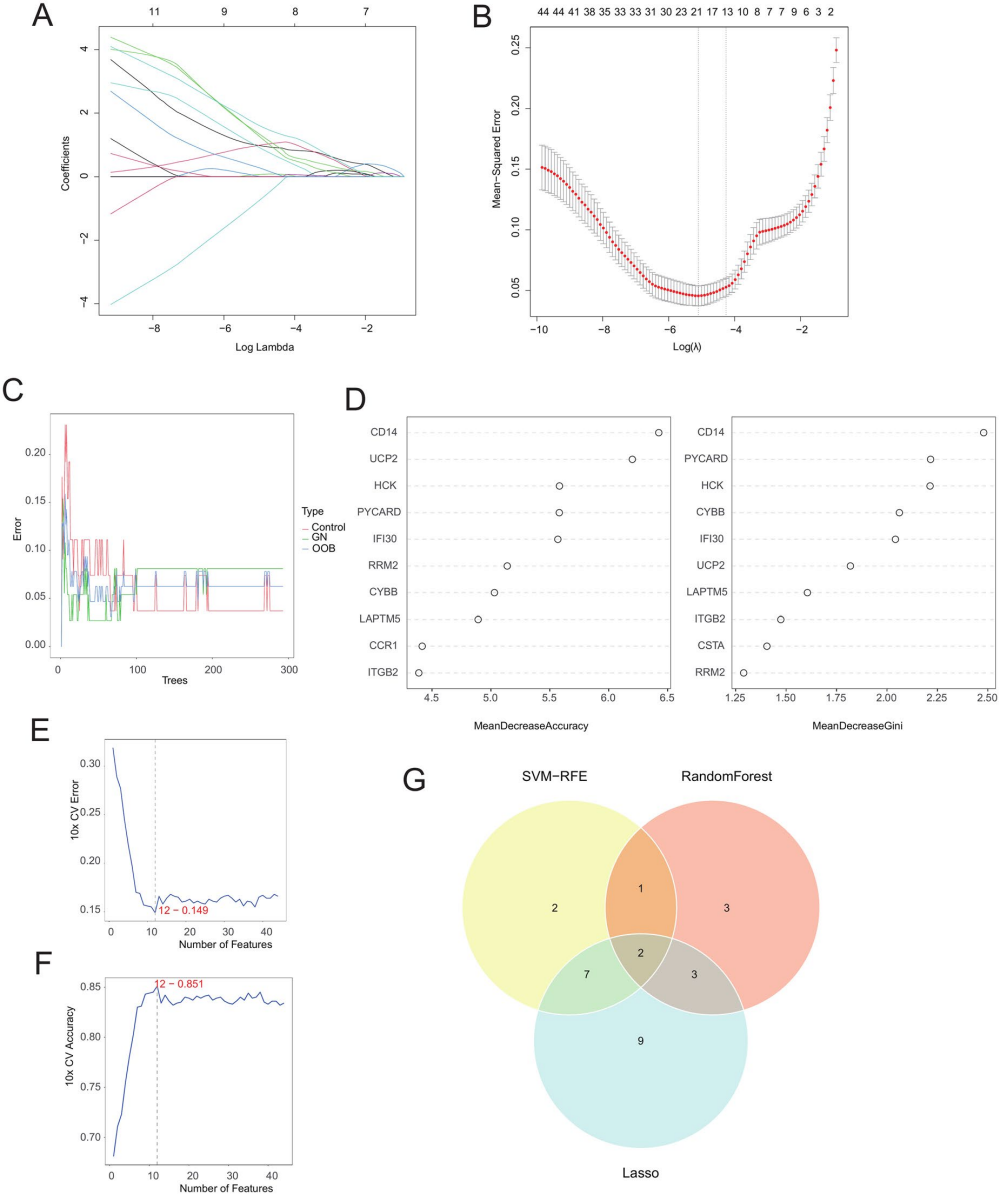
Identification of candidate diagnostic biomarkers for ANCA-GN using machine learning approaches. (A) The change trajectory of independent variables in LASSO regression, where the x-axis represents the logarithm of the variable lambda, and the y-axis represents the corresponding coefficients. (B) The confidence interval for each lambda in LASSO regression. (C) The error rate in random forest analysis compared to the number of classification trees. (D) The top 10 key genes ranked by two importance measures in the random forest algorithm. (E) The error rate curve for the SVM-RFE method. (F) The accuracy curve for the SVM-RFE method. (G) The intersection of key genes identified by the three machine learning methods to determine the Hub genes.

We further applied LASSO regression and random forest algorithms to identify key genes. Through LASSO regression analysis, we selected 21 key genes (Figure 8A,B). Using the random forest algorithm, based on feature importance measured by Mean Decrease Accuracy (MDA) and Mean Decrease Gini (MDG), we identified 9 key genes (Figure 8C,D). Using the SVM-RFE method, we selected 12 key genes (Figure 8E,F). Finally, the intersecting key genes identified by each method yielded 2 most critical genes as hub genes, *PYCARD* and *IFI30* (Figure 8).

### Diagnostic value of hub genes

**Figure 9.**
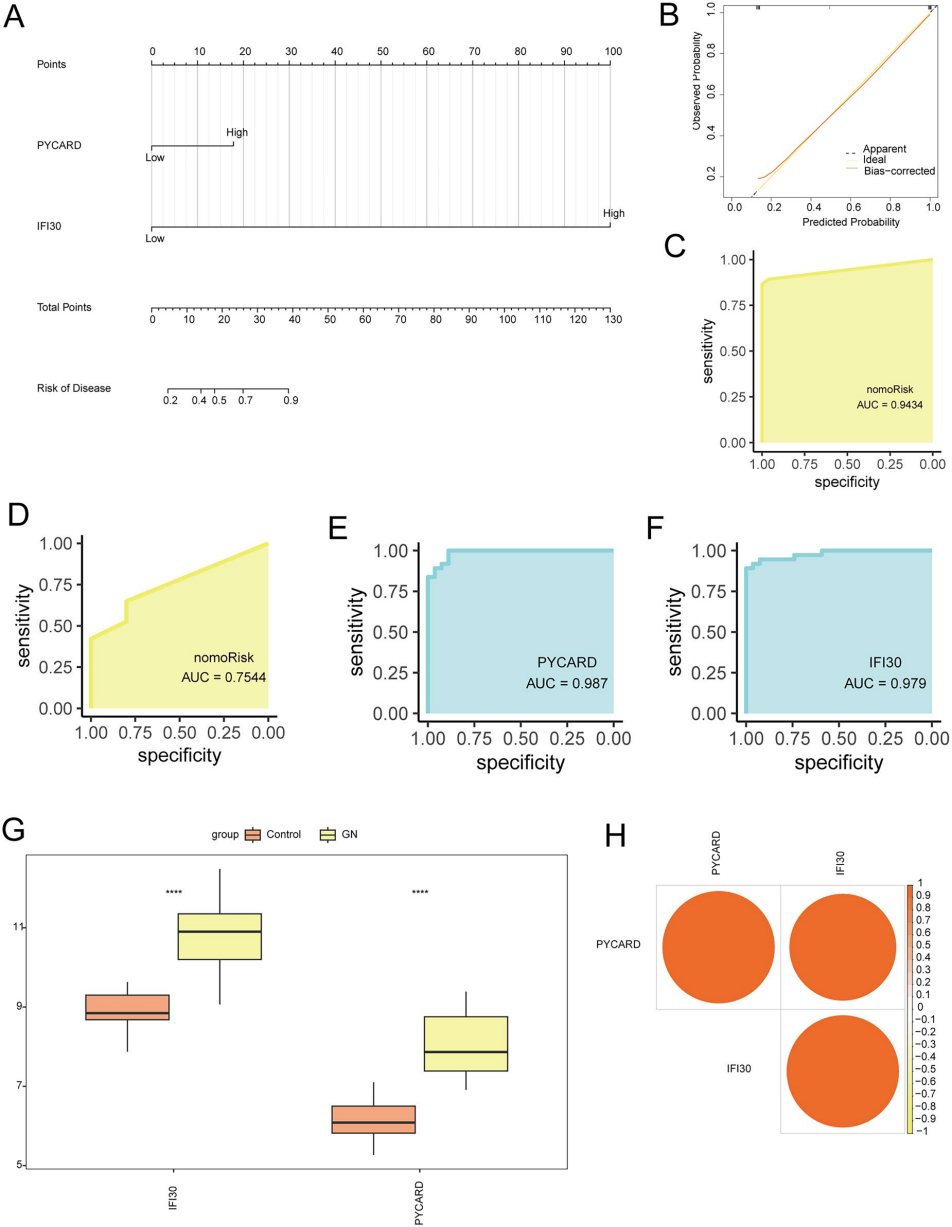
Nomogram and expression validation of hub genes. (A) Nomogram for ANCA-GN diagnosis. (B) Standard curve of the nomogram. (C) ROC curve of the nomogram model. (D) ROC curve validation of the nomogram model using an external dataset. (E) ROC curve for *PYCARD.* (F) ROC curve for *IFI30*. (G) Boxplot of hub gene expression. (H) Correlation heatmap of hub genes. Asterisks indicate *p*-values: ****p < 0.0001, ***p < 0.001, **p < 0.01, *p < 0.05. ROC: Receiver Operating Characteristic.

We constructed a diagnostic nomogram model for ANCA-GN using the feature genes (*PYCARD* and *IFI30)* (Figure 9A) and evaluated its predictive ability with a calibration curve. The calibration curve showed minimal deviation between the true ANCA-GN risk and the predicted ANCA-GN risk, indicating that the ANCA-GN model is highly accurate (Figure 9B). ROC curve analysis further confirmed the model’s validity (ROC > 0.9, Figure 9C). ROC curve analysis using the external dataset GSE108112 also confirmed the model’s correctness (ROC > 0.7, Figure 9D).

To further validate the diagnostic value of the hub genes, we performed ROC curve analysis for each hub gene. The AUC values for *PYCARD* (AUC = 0.987) and *IFI30* (AUC = 0.979) were both greater than 0.9 (Figure 9E,F), indicating that these hub genes have strong discriminatory potential as biomarkers for ANCA-GN.

We also analyzed the expression patterns of the key genes between the ANCA-GN and control groups using box plots and correlation heatmaps. The expression levels of the two hub genes were significantly higher in the ANCA-GN group compared to the control group (Figure 9G), and there was a strong positive correlation between the hub genes (Figure 9H).

### Immune infiltration

**Figure 10.**
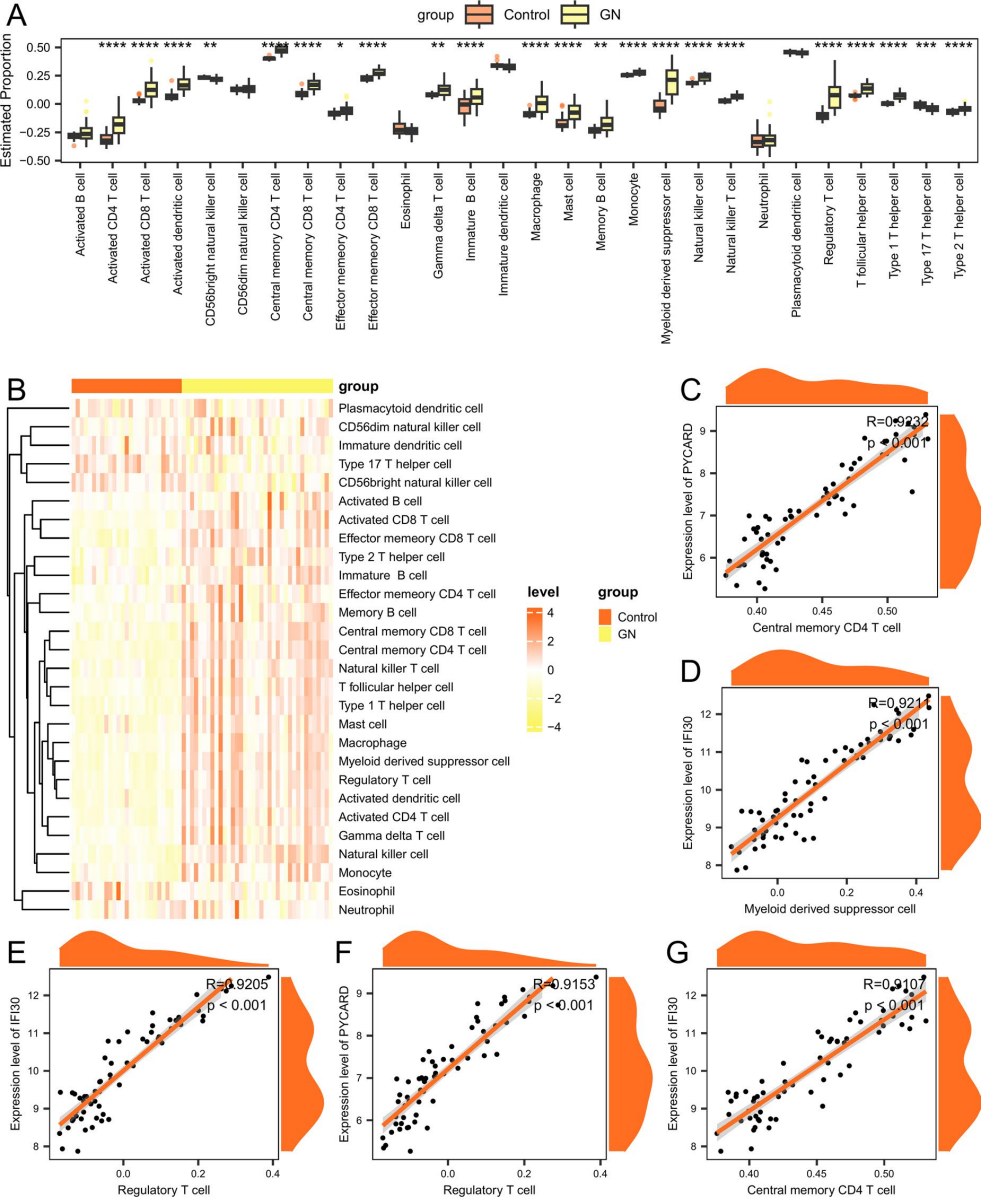
Differences in immune infiltration between ANCA-GN and control groups. (A) Differences in the estimated immune cell infiltration proportions between the ANCA-GN and control groups. (B) Heatmap showing changes in immune infiltration levels between the ANCA-GN and control groups. (C) Correlation scatter plot between *PYCARD* and Activated CD8+ T cell. (D) Correlation scatter plot between *IFI30* and Activated CD8+ T cell. (E) Correlation scatter plot between *PYCARD* and Central memory CD8+ T cell. (F) Correlation scatter plot between *IFI30* and Central memory CD8+ T cell. (G) Correlation scatter plot between *PYCARD* and Effector memory CD8+ T cell. Asterisks indicate *p*-values: ****p < 0.0001, ***p < 0.001, **p < 0.01, and *p < 0.05.

Immune cell infiltration might play an important role in the pathogenesis of ANCA-GN. Therefore, we investigated the correlation between ANCA-GN/control samples and infiltrating immune cells. Among 28 immune cell types, 22 showed significant differences in immune cell infiltration abundance between the two groups (*p* < 0.05) (Figure 10A, Supplementary Table 13). Specifically, 20 immune cell types (Supplementary Table 14) had significantly higher infiltration levels in the ANCA-GN group compared to the control group (Figure 10A).

As shown in Figure 10(B), the overall immune cell infiltration levels exhibited large differences between the ANCA-GN and control groups. We also examined the significant correlations between each hub gene and the corresponding immune cells. The top five correlation scatter plots are displayed. *PYCARD* was significantly correlated with Activated CD8+ T cell (*R* = 0.679, *p* < 0.001) (Figure 10C); *IFI30* was significantly correlated with Activated CD8+ T cell (*R* = 0.628, *p* < 0.001) (Figure 10D); *PYCARD* was significantly correlated with Central memory CD8+ T cell (*R* = 0.873, *p* < 0.001) (Figure 10E); *IFI30* was significantly correlated with Central memory CD8+ T cell (*R* = 0.878, *p* < 0.001) (Figure 10F); *PYCARD* was significantly correlated with Effector memory CD8+ T cell (*R* = 0.771, *p* < 0.001) (Figure 10G).

**Figure 11.**
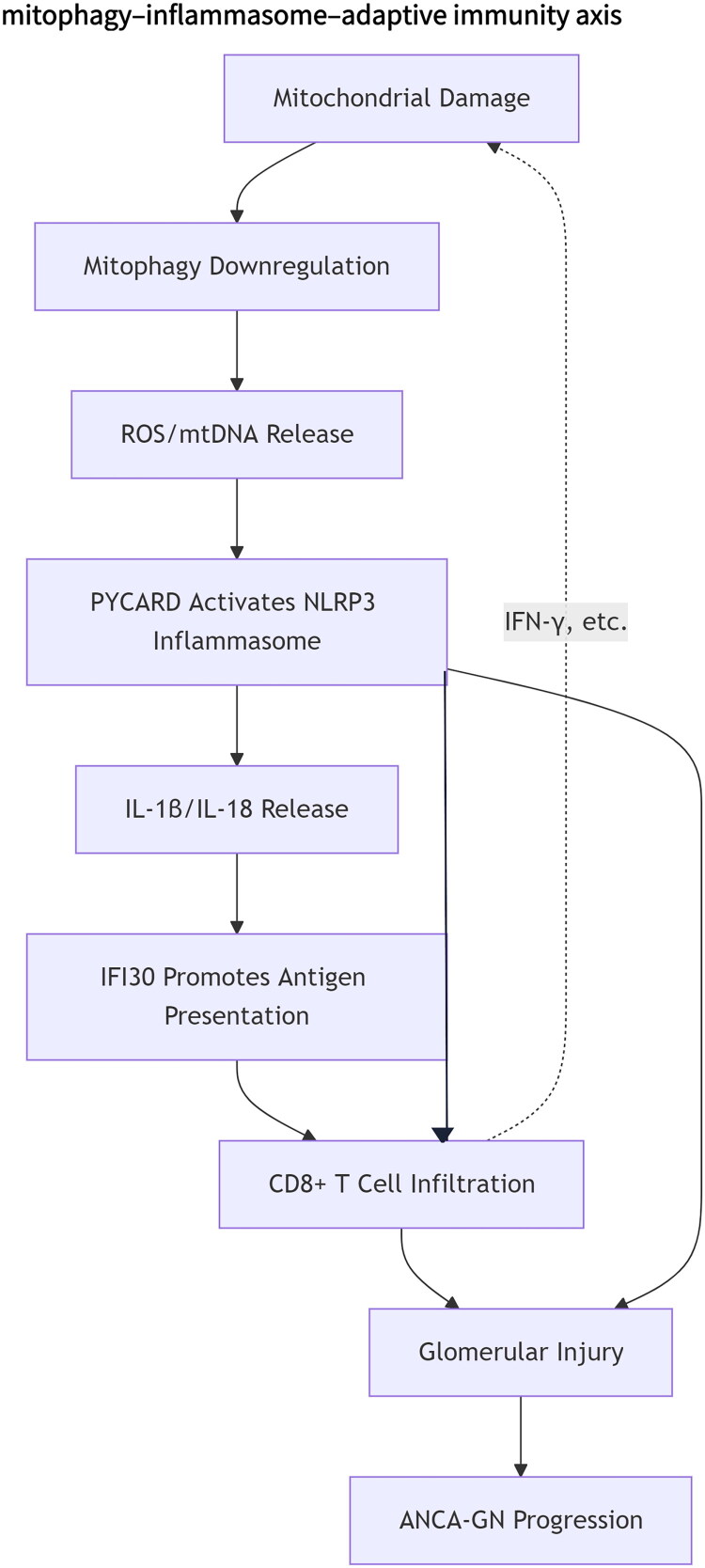
Mitophagy–inflammasome–adaptive immunity axis. ANCA-GN: ANCA-associated glomerulonephritis

Based on transcriptomics, bioinformatics, and existing literature, we constructed a concise mechanistic flowchart (Figure 11 mitophagy–inflammasome–adaptive immunity axis).

## Discussion

ANCA-GN is a severe autoimmune kidney disease characterized by pauci-immune necrotizing crescentic glomerulonephritis, which often progresses rapidly to kidney failure [1]. Despite advancements in treatments such as immunosuppressive agents or biologics, a considerable proportion of patients still experience refractory or recurrent cases [47]. This study utilized integrative bioinformatics methods, combining transcriptomic data and various machine learning algorithms, to identify molecules associated with mitophagy and immunity in ANCA-GN: *PYCARD* and *IFI30*. These hub genes demonstrate excellent diagnostic performance and are closely related to CD8+ T cell infiltration. These findings not only reveal the molecular heterogeneity of ANCA-GN but also propose a new model for its pathogenesis, involving the mitophagy-inflammasome-immune axis.

Recent studies show that mitophagy is highly active in renal tubular epithelial cells, regulating ROS clearance and energy metabolism (such as the balance between oxidative phosphorylation and glycolysis) *via* pathways like BNIP3 and FUNDC1,abnormal regulation of mitophagy is closely linked to diseases like kidney fibrosis and AKI [48,49]. However, recent evidence also highlights the importance of mitophagy in glomerular lesions, as mitochondrial defects in podocytes, mesangial cells, and macrophages in diseases such as lupus nephritis and diabetic kidney disease (DKD) can trigger oxidative stress, cytoskeletal damage, and inflammation [9,50,51]. We hypothesize that in ANCA-GN, mitochondrial dysfunction may exacerbate glomerular damage by promoting oxidative injury and inflammation signaling in both resident and infiltrating immune cells. This study focuses on the glomerular region, and through consensus clustering of mitophagy-related genes, we successfully classified ANCA-GN patients into two subtypes: cluster_1 and cluster_2. A total of 131 DEGs were identified, with 126 upregulated and 5 downregulated genes in cluster_1 compared to cluster_2. This further confirms the key role of mitochondrial quality control mechanisms in immune-driven ANCA-GN glomerular lesions.

Among the subtype-specific DEGs, PYCARD and IFI30 emerged as key participants. PYCARD is known as the core adaptor of the NLRP3 inflammasome and facilitates the binding of NLRP3 inflammasome, promoting caspase-1-dependent pyroptosis and neutrophil extracellular trap (NET) formation [17,52]. Some studies have reported that PYCARD can participate in the regulation of mitophagy *via* the inflammasome pathway [53]. Other research has suggested that PYCARD expression is positively correlated with NOD-like receptor signaling pathways, promoting immune cell infiltration [54]. PYCARD has also been identified as a core apoptosis gene in osteoarthritis macrophages [25]. Currently, there are few direct studies on PYCARD in glomerular diseases, and this study links PYCARD overexpression with ANCA-GN subtyping, suggesting its crucial role in driving glomerular injury through inflammasome mediation and immune cell infiltration.

Meanwhile, IFI30, a lysosomal thiol reductase essential for MHC II antigen processing, plays a role in antigen presentation [21]. Studies have shown that IFI30 is associated with various cancers, including breast, kidney, and prostate cancers, primarily through the regulation of antigen processing and immune infiltration in tumor progression [55–57]. Other studies have suggested that IFI30 is associated with autoimmune diseases such as systemic lupus erythematosus and Sjögren’s syndrome, where it may affect antigen presentation or macrophage polarization, contributing to abnormal immune activation [58,59]. Although few studies have reported the association between IFI30 and ANCA-GN, this study suggests that IFI30’s role in the glomerulus of ANCA-GN may promote the presentation of autoantigens (e.g., MPO and PR3) or facilitate macrophage polarization, thereby exerting its molecular function and ultimately linking lysosomal function with adaptive autoimmunity. The co-upregulation of PYCARD (innate immunity) and IFI30 (adaptive immunity) suggests a potential collaborative pathway, making these two genes potential targets for subtype-specific therapies.

Pathway enrichment analysis further validated this mechanistic framework. DEGs were enriched in immune and inflammatory pathways, including Toll-like receptor signaling, the intestinal immune network for IgA production, and cytokine-cytokine receptor interactions, all of which are closely related to neutrophil activation, NETosis, and ANCA generation [60,61]. Conversely, metabolic pathways like cytochrome P450, inositol phosphate metabolism, and glycosphingolipid biosynthesis were downregulated, suggesting impaired metabolic adaptation and potential glucocorticoid resistance [62,63]. These findings collectively outline the integrative mechanism of the mitophagy-inflammasome-immune-lysosome axis, providing important clues for the pathophysiology of ANCA-GN.

Immune infiltration analysis showed widespread activation of both innate and adaptive immune cells in ANCA-GN, with particularly significant enrichment of CD8+ T cell subsets. Studies have shown that after activation of the NLRP3 inflammasome by PYCARD, IL-1β/IL-18 secretion promotes CD8+ T cell differentiation into effector/memory phenotypes [64]. Additionally, IFI30’s role in MHC-II antigen processing directly activates CD8+ T cells, a mechanism confirmed in prior studies [65,66]. Notably, our study found a novel and highly specific association between PYCARD and IFI30 and activated/central memory CD8+ T cells, a finding that has not been reported in ANCA-GN before. Studies suggest that central memory CD8+ T cells are long-lived mediators of relapsing autoimmunity, potentially providing a mechanistic basis for ANCA-GN recurrence [67]. Research has shown that scRNA-seq analysis indicates PYCARD is primarily expressed in myofibroblasts and macrophages within the glomerular immune microenvironment, along with Foxp3+ regulatory T cell infiltration [68]. Furthermore, scRNA-seq analysis confirmed that IFI30 is primarily localized to monocyte/macrophage populations, strongly correlating with immune cell infiltration [66]. These independent datasets support our hypothesis that PYCARD and IFI30 may regulate the activation and differentiation of T cells in the glomerular immune microenvironment. However, future single-cell or spatial transcriptomic studies are required to validate this conclusion in ANCA-GN.

The diagnostic model constructed from PYCARD and IFI30 demonstrated excellent predictive accuracy (AUC = 0.94 in discovery, 0.75 in validation), suggesting robust clinical potential. However, it is important to note that these results are specific to ANCA-GN and have not been validated in other types of glomerulonephritis (e.g., IgA nephropathy and membranous nephropathy). Future studies should explore whether these genes also show abnormal expression in other immune-mediated glomerular diseases. The excellent diagnostic performance of PYCARD and IFI30 supports their potential as kidney biopsy biomarkers for ANCA-GN. Going forward, the development of liquid biopsy technologies to detect PYCARD and IFI30 in noninvasive samples (e.g., urine) could enable early screening, subtyping, and dynamic monitoring of therapeutic efficacy, thus supporting precision medicine in ANCA-GN management.

As a core component of the NLRP3 inflammasome, PYCARD upregulation underscores the importance of the NLRP3 pathway in ANCA-GN pathogenesis. Recently, various NLRP3 inhibitors (e.g., MCC950, dapansutrile) have shown promising results in autoimmune and inflammatory diseases [69]. Preliminary animal and clinical studies suggest these inhibitors can suppress renal inflammation, indicating that NLRP3-targeted therapies may be a new strategy for precision treatment of ANCA-GN [70]. IFI30 plays a key regulatory role in antigen presentation, influencing the body’s autoimmune response. Innovative drugs targeting the IFI30-dependent process, such as GILT inhibitors, are under development and may offer personalized immune intervention [66]. These developments expand new directions for immune modulation in ANCA-GN treatment.

This study has several limitations. First, the small sample size and potential batch effects between different datasets may lead to result variability. Second, the study only analyzed bulk transcriptomic data without performing single-cell or spatial transcriptomic analyses. Third, the pathogenicity of the identified genes has not been confirmed—observed changes may reflect secondary inflammatory responses rather than causal mechanisms. Fourth, the expression levels and diagnostic performance of PYCARD and IFI30 have not been evaluated in other types of glomerulonephritis, which limits the generalizability of our findings. Future research should include experimental and clinical validation to clarify the causal relationships and functional significance of these genes in ANCA-GN.

In conclusion, this integrative transcriptomic and machine learning study identifies PYCARD and IFI30 as novel immune-lysosomal biomarkers of ANCA-GN. It uncovers a mitochondrial mitophagy-inflammasome-adaptive immune network that may explain disease heterogeneity and progression mechanisms. These molecular mechanisms provide a foundation for precision diagnosis and targeted treatment of ANCA-GN. Future studies should combine multi-omics integration, clinical sample validation, and new drug development to promote the clinical translation of molecular biomarkers and targeted interventions, ultimately improving patient prognosis.

## Supplementary Material

Supplementary Materials WGCNA.doc

## Data Availability

All data relevant to this study are included in the main article and its supplementary materials. All supplementary materials have been uploaded to Zenodo, and the corresponding link is https://doi.org/10.5281/zenodo.18084498. The datasets used to support the conclusions of this work are publicly available in the Gene Expression Omnibus (GEO) database (https://www.ncbi.nlm.nih.govgeo/).

## Code availability statement

The R code used for some of the analysis workflows in this study has been organized and uploaded to Zenodo. The unique DOI for the code is: 10.5281/zenodo.17519865, which can be independently cited and reused by other researchers in the field.
