# Supplementary material for: Integrative transcriptomic and machine learning analysis identifies PYCARD and IFI30 as immune-lysosomal biomarkers of ANCA-associated glomerulonephritis
Source: Ren Fail. 2026 Feb 10;48(1):2624286. doi: 10.1080/0886022X.2026.2624286 (PMC12895877; doi:10.1080/0886022X.2026.2624286)
Supplement: Supplementary Materials WGCNA.doc [file IRNF_A_2624286_SM6285.doc]

**Supplementary Materials for network construction details**

**Weighted Gene Co-expression Network Analysis (WGCNA)and Key Module Identification**

The WGCNA algorithm was implemented using the R package "WGCNA" (version 1.70-3) to construct co-expression networks. Pearson correlation coefficients were calculated to assess similarity between gene expression profiles, and a power function (β=12) was applied to achieve scale-free topology. The R function "PickSoftThreshold" was used to determine the optimal soft threshold. Gene modules, representing clusters of densely interconnected genes, were identified via hierarchical clustering and dynamic tree cutting. Topological overlap matrices (TOM) were generated to quantify module similarity. Module eigengenes (MEs), representing the first principal component of each module, were correlated with mitophagy activity using Pearson analysis. Modules significantly associated with mitophagy were selected for further analysis. Co-expression network structures were visualized using heatmaps of topological overlap.
